# Sensors for Cryogenic Isotope-Separation Column

**DOI:** 10.3390/s20143890

**Published:** 2020-07-13

**Authors:** Eva H. Dulf, Clement Festila

**Affiliations:** 1 Department of Automation, Faculty of Automation and Computer Science, Technical University of Cluj-Napoca, Memorandumului Str. 28, 400014 Cluj-Napoca, Romania; Clement.Festila@aut.utcluj.ro; 2 Physiological Controls Research Center, Óbuda University, H-1034 Budapest, Hungary

**Keywords:** cryogenic isotope-separation, capacitive liquid-level sensor, pulse width modulation (PWM) electrical power sensor, system monitoring

## Abstract

Cryogenic isotope-separation equipment is special, encountered in relative few research centers in the world. In addition to the main equipment used in the operation column, a broad range of measuring devices and actuators are involved in the technological process. The proper sensors and transducers exhibit special features; therefore, common, industrial versions cannot be used. Three types of original sensors with electronic adapters are presented in the present study: a sensor for the liquid carbon monoxide level in the boiler, a sensor for the liquid nitrogen level in the condenser and a sensor for the electrical power dissipated in the boiler. The integration of these sensors in the pilot equipment is needed for comprehensive system monitoring and control. The sensors were tested on the experimental equipment from the National Institute for Research and Development of Isotopic and Molecular Technologies from Cluj-Napoca.

## 1. Introduction

An important research activity of physicists and chemists is dedicated to isotopes—stable or radioactive. Isotopes are species of chemical elements with the same number of protons and electrons in the nucleus, but with a different number of neutrons in nucleus. For any chemical element, the natural distribution of isotope concentration is constant, being a particular property of each element. For example, in the case of carbon (C), the basic isotope (^12^C) has a concentration of 98.89%, while the most important heavier isotope (^13^C) has an abundance of 1.11%.

Research for new isotope properties and for processing methods—and, consequently, the extension of the isotope application range—has led to new and more efficient equipment for the isotope-separation process. Starting from the natural abundance of the isotope of interest, during the separation process it is possible to increase the isotope concentration to a desired level. Various chemical compounds based on enriched (^13^C) have a broad field of application [1]:

- Applications in medical fields: in clinical studies, differential diagnosis of multiple diseases, state of illness judgment, treatment evaluation, research on organ function and development of new drugs [2], such as PET diagnosis [3], ^13^C-urea breath test [4], tumor therapy (boron neutron capture therapy) research on drugs [5], etc.;
- Applications in life sciences fields: in the spectrum research of the structure and function of protein species by nuclear magnetic resonance (NMR) and mass spectrometer (MS), including isotope-coded affinity tag testing method (ICATTM), stable isotope labeled amino acid cell cultivation (SILAC), absolute quantification protein analysis method (AQUATM) [6];
- Applications in energy metabolism: in sports medicine, child nutrition, food nutrition, weight loss and diet for astronaut using he-isotope tracing method [7];
- Applications in agricultural scientific research fields: in the research of plant physiology and biochemistry, nutrition of soil and plant, plant protection, the improvement of rice, flower, agricultural products and other crops, nitrogen cycle of grassplot [8];
- Applications in the research of environmental science: monitoring of ecosystem pollution, determining of the length of food chains, ascertaining the pollutant sources in air and water and defining the distribution area of plant [9,10];
- Applications in analysis and testing: in the testing of food [11], pesticide residue analysis [12], drug and excitant, heroin testing [13], also in the research of geology, geochemistry, paleontology and ecology [14,15].

Many principles, procedures and methods are known and used today to separate one or more isotopes from the “basic species” [16]. Some may be used only for laboratory research, while others are proper for industrial large-scale developments.

The National Institute for Research and Development of Isotopic and Molecular Technologies (NIRDIMT) in Cluj-Napoca, Romania is recognized over the world for scientific results in the production of carbon, oxygen, nitrogen and other isotopes [17]. There are relatively few scientific international research centers known in this domain, which disseminates particular results and presents possible isotope applications domains. The technology, equipment data and structure, details about the mode of operation for isotope-separation equipment are still “classified”. In these conditions, the research team from NIRDIMT conceived, developed, designed and built various isotope-separation equipment. Our goal is to design and implement a proper monitoring system for these equipment, the first case study being the (^13^C) isotope-separation column. Being a unique process, with particular properties, market available sensors are not suitable for use.

The main objective of the present work is to present the three dedicated sensors, designed and implemented for this cryogenic isotope-separation equipment.

The study is organized as follows: After this short introductory section, Section 2 presents the cryogenic separation column. Section 3 deals with the dedicated sensors. Section 4 details the corresponding practical problems. The work ends with a results and conclusion section.

## 2. The Cryogenic Separation Column

**Figure 1:**
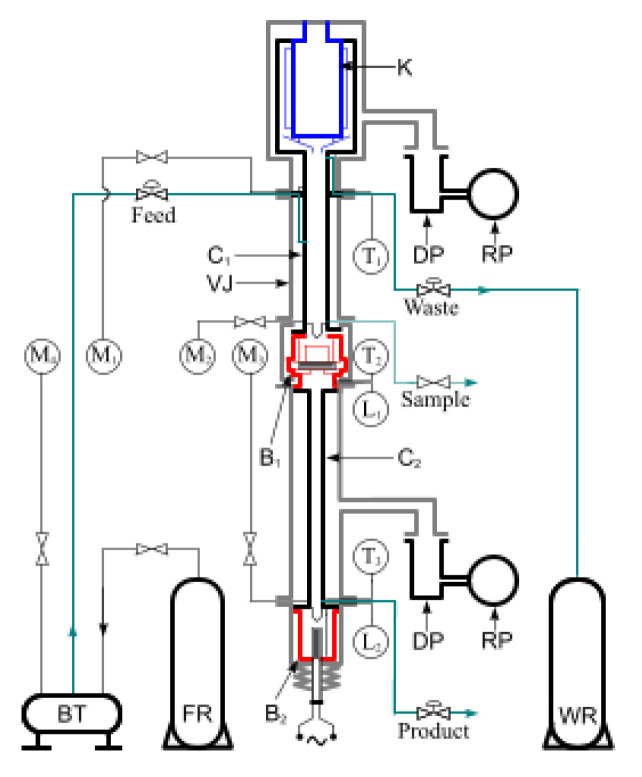
Isotope-separation column scheme, with condenser K, primary column C1, final column C2, reboilers B1–B2, vacuum jacket VJ, rough pump RP, diffusion pump DP, temperature sensors T1–T3, manometers M1–M4, level sensors L1–L2, feed reservoir FR, buffer tank BT, waste reservoir WR.

**Figure 2:**
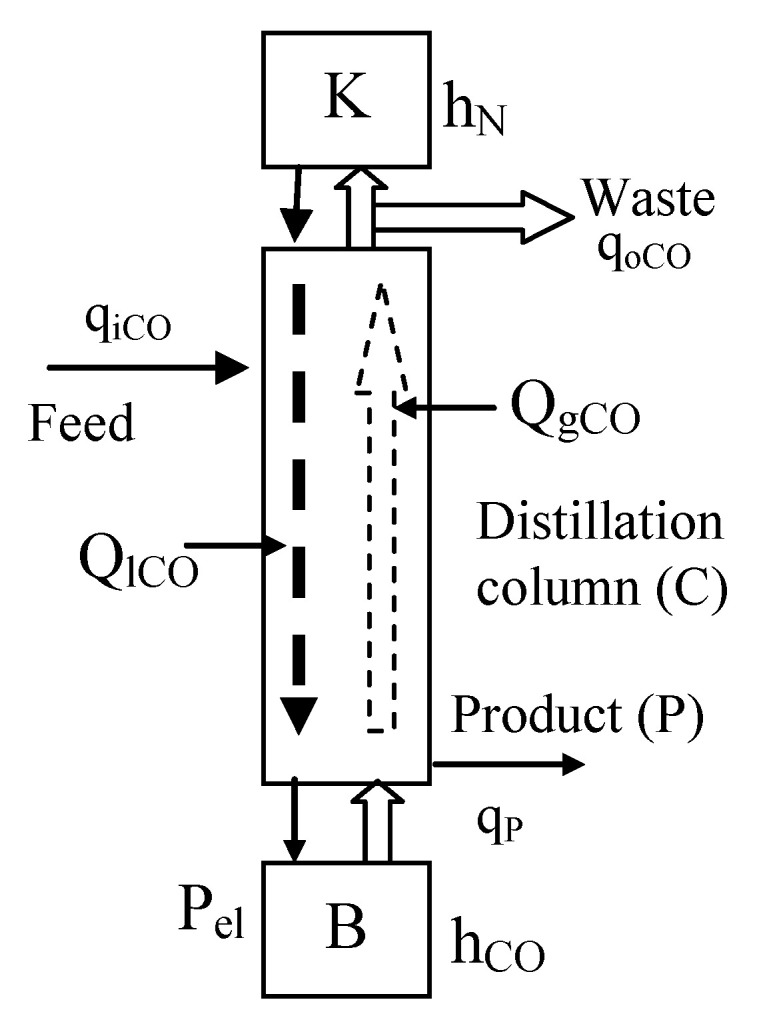
Isotope-separation column simplified scheme, with condenser K, column C, boiler B, input (CO) flow q_iCO_, waste flow q_oCO_, product flow q_P_. internal gaseous flow Q_gCO_, internal liquid flow Q_lCO_, electrical power P_el_, (N) level in the condenser h_N._

Unfortunately, the physical and chemical properties of the compounds of (^12^C) and (^13^C) are very close, hence the difficulty of “separation”. Even though, these small property differences must be used to increase the abundance in (^13^C). By the cryogenic separation process, at very low temperature (~−190 °C), carbon monoxide (^12^CO) has a higher vapor pressure (${\overset{o}{p}}_{12CO}$) than the pressure (${\overset{o}{p}}_{13CO}$) of the compound (^13^CO), in accordance to the ratio [18,19]:

(1) $$\alpha =\frac{{\overset{o}{p}}_{12CO}}{{\overset{o}{p}}_{13CO}}>1,\;\alpha \approx 1.01$$

where (*α*), the “elementary separation coefficient” is essential for the separation process efficiency. If the gaseous and liquid phases of the carbon monoxide are in contact, the chemical compound (^13^CO) accumulates in the liquid phase as the end-product, while (^12^CO) accumulates in the gaseous phase, which must be evacuated as waste. The separation efficiency increases by the multiplication (*α*^k^) of the (^13^C) exchange process in the isotope-separation column in continuous recirculation (Figure 1) where the gaseous carbon monoxide upstream is in contact with the liquid downstream of carbon monoxide [19]. A simplified scheme is presented in Figure 2, used for the corresponding equations description.

The internal flows of gaseous and liquid phases—the column loads—are generated by thermal effect. The electrically heated boiler (B), from the column basis, generates by evaporation the gaseous ascendant phase in the column, while the condenser (K) from the top side of the column, cooled by liquid nitrogen, Figure 2, converts the gaseous phase to a liquid phase downstream. In order to increase the contact surface between gas and liquid, in column is fixed a special packing material [20,21].

During the normal operation, the column is supplied with gaseous carbon monoxide at the in-flow (q_iCO_) and the waste is evacuated by the flow (q_oCO_).

The main variables related to the column operation may be divided into control (manipulated) variables, internal measurable variables, internal unmeasurable variables and external disturbances.

The control (manipulated) variables are:

- q_iCO_ = carbon monoxide inflow (mol/sec);
- q_oCO_ = carbon monoxide outflow (mol/sec);
- P_el_ = electrical power developed in the boiler (W);
- h_N_ = liquid nitrogen level in the condenser (cm).

The internal measurable variables are:

- p_B_, p_m_, p_k_ = the pressure in the boiler, middle region and in the condenser vicinity (mm H_2_O);
- h_CO_ = carbon monoxide liquid level in the boiler (mm);
- concentration (c_p_) of (^13^C) in the liquid phase from boiler (mol/m^3^).

The internal unmeasurable variables:

- Q_lCO_, Q_gCO_ = liquid downstream and gaseous upstream of carbon monoxide (mol/sec/cm^2^)
- H_b_, H_p_, H_c_, H_g_, H_w_ = the retained carbon monoxide material, called the “hold-up” values, respectively in the boiler, on the packing material, on the condenser wall, in the gaseous phase and on the column wall (mol).

It can be appreciated that (H_b_, H_p,_ H_c_) have much greater values than (H_g_ + H_w_), so that (H_g_ + H_w_) can be considered neglectable. The total column hold-up is [22,23]:

(2) $$H_{\Sigma }{=H}_{b}{+H}_{p}{+H}_{c}$$

which approximates the total quantity of carbon monoxide from column.

The external disturbances are:

- the under-pressure in thermal insulation system (vacuum jacket, p ≈ 10^−5^ Torr);
- the external (ambient) temperature (°C);
- the final product extract material flow (q_p_) (mol/h).

Due to the fact that q_p_ is small in comparison with (q_iCO_, q_oCO_), in this study (q_p_) is neglected (q_p_ ≈ 0).

The system monitoring and control must deal with the key variables of the equipment. To find these, we need the basic equations of the column.

In a simplified description of the cryogenic normal mode of operation, the liquid carbon monoxide volume, accumulated in the boiler (V_B.CO_), Figure 2, is given by the equation:

(3) $$V_{B.\mathrm{CO}}=\int_{0}^{t}\left(q_{\mathrm{iCO}}\left(\tau \right)-q_{o\mathrm{CO}}\left(\tau \right)\right)d\tau +\int_{0}^{t}\left(q_{k}\left(\tau \right)-q_{B}\left(\tau \right)\right)d\tau -\Delta H_{p}$$

where (q_k_) is the condensation flow, (q_B_) is the evaporation flow and (ΔH_p_) is the “hold-up” variations on the packing material.

The linear dependences of the main variables can be presented as:

(4) $$\begin{array}{l} q_{k}=k_{N}\cdot h_{N}\\ q_{B}=k_{el}\cdot P_{el}\\ \Delta H_{p}=k_{p}\left(p_{B}-p_{k}\right) \end{array}$$

with (k_N_), (k_el_) and (k_p_) specific constants. It can be concluded that $V_{B.CO}=h_{B}\cdot S_{B}$, where (S_B_) is the boiler cylindrical crossover section and (h_B_) is the (CO) level in the boiler. For the liquid (CO) level results:

(5) $$h_{B}=\frac{1}{S_{B}}\int_{0}^{t}\left(q_{iCO}\left(\tau \right)-q_{oCO}\left(\tau \right)\right)d\tau +\int_{0}^{t}\frac{k_{N}}{S_{B}}\cdot h_{N}\left(\tau \right)d\tau -\int_{0}^{t}\frac{k_{el}}{S_{B}}\cdot P_{el}\left(\tau \right)d\tau -\frac{k_{p}}{S_{B}}\cdot \left(p_{B}-p_{k}\right)$$

This equation emphasizes the key variables involved in the column operation besides the flows (q_iCO_, q_oCO_):

- the liquid carbon monoxide level in the boiler (h_B_),
- the liquid nitrogen level in the condenser (h_N_)
- and the electrical heating power (P_el_).

For column monitoring and control purposes, the authors designed and implemented three sensors for these key variables: the first is based on a cylindrical capacitive sensor for providing information about the liquid carbon monoxide level in the boiler; the second capacitive sensor provides information about the liquid nitrogen level in the condenser; and the third one—an electronic power sensor—provides information about the thermal power dissipated in the boiler. Because the isotope separation is particular equipment operating in cryogenic conditions, classical sensors cannot be embedded in the column design, due to placement, shape, operation and even dimensions issues. The insulated material used for electrical connections to transducers working at about −193 °C, also raises special problems.

## 3. Sensor System for Cryogenic Column Control

The pressures in the boiler (p_B_) and the condenser (p_k_) can be measured with high accuracy using common low-pressure transducers [24,25] with or without electrical output signal.

The carbon monoxide inflow (q_iCO_) and outflow (q_oCO_) may be measured and controlled by automatic controlled mechanical-electric valves [26], available on the market. The particular difficulty in this case study is the very low value of the processed flow, so that the desired balance (q_iCO_-q_oCO_) ≈ 0 cannot be achieved accurately for long term operation. If the liquid nitrogen level (h_N_) and the electrical power (P_el_) are constant, the flow unbalance is sensed by the liquid carbon monoxide level (h_B_) in the boiler and the flow (q_iCO_) or (q_oCO_) must be periodically corrected.

### 3.1. Sensor for Liquid Carbon Monoxide Level in the Boiler

The necessity to know the accurate value of the (CO) level in the boiler (h_B_) is relevant for many reasons. A minimum, but constant value of (h_B_) is a practical measure for the high efficiency of the isotope-separation process [20]. The evolution of the liquid (CO) level also gives information about the stability of the column operation and about the stability margin up to an unstable disastrous situation: column “flooding” [20]. It was tested a particular, intricate column construction [20] with a special small glass window in the basis of the column in order to view the liquid level in the boiler. Due to the generated huge technical problems, this solution was abandoned.

**Figure 3:**
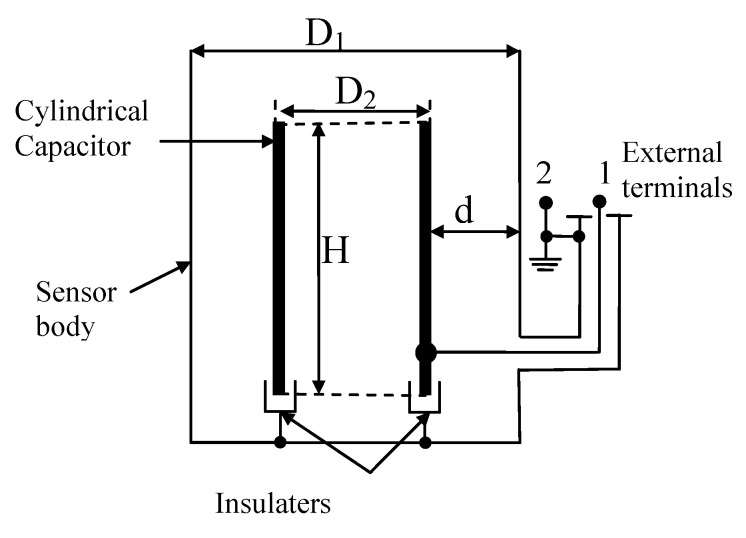
Scheme of the liquid (CO)-level sensor in the boiler.

Although many versions of liquid-level sensors are available [24,25,27,28], the constructive particularities of the separation column impose particularized sensors. The place, shape and dimension of the liquid carbon monoxide-level sensor is an important element embedded in the isotope-separation column design. The authors propose the use of a capacitive sensor—based on the difference between the liquid (ε_l_) and gaseous (ε_g_) permittivity coefficients. It is a cylindrical capacitor embedded in the boiler (column body), with the height (H) and the external/internal diameters (D_1_/D_2_). This sensor is schematically presented in Figure 3.

If the liquid level is (h), three equivalent capacitors are parallel connected, so that the total capacity (C_Σ_) is:

(6) $$C_{\Sigma }=C_{L}+C_{g}+C_{p}$$

Starting from the general equation of the cylindrical capacitor:

(7) C=2π(εr·εo)·1ln(D1D2)·H=2πεoln(D1D2)·εr·H

results:

$C_{l}=\varepsilon_{l}\cdot k_{a}\cdot h$ (F) for liquid level

$C_{g}=\varepsilon_{g}\cdot k_{a}\cdot \left(H-h\right)$ (F) for “gaseous” level

$C_{p}≅\mathrm{constant}$ for the parasitic capacitor (pF)

with ka=2πε0ln(D1D2).

Introducing these results in Equation (6), we have

(8) $$C_{\Sigma }=k_{a}\left(\varepsilon_{l}-\varepsilon_{g}\right)\cdot h+k_{a}\cdot \varepsilon_{g}\cdot H+C_{p}$$

so that

(9) h=1ka(εl−εg)·CΣ−Cpka+εg·H(εl−εg)=γ·CΣ−β

Equation (9) describes the basic operational principle of the sensor, with (γ, β) constants.

**Figure 4:**
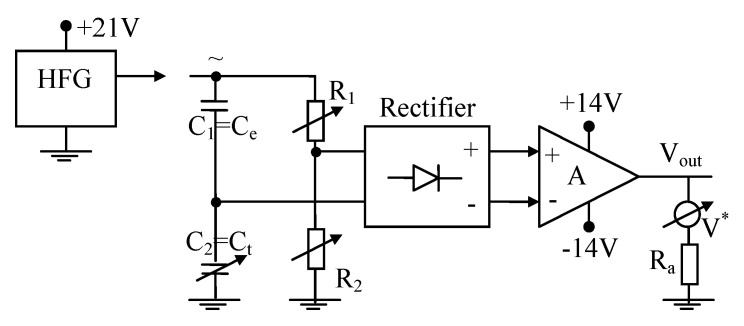
Electronic scheme of the adapter for liquid CO-level transducer.

The output (useful) signal of the transducer is generated by the electronic adapter, Figure 4, where a high-frequency generator (HFG) (f ~300 kHz) supplies a mixed (R/R) and (C/C) bridge. In the (RC) bridge, (C_1_) is a reference capacitor and (C_2_ = C_Σ_) is the transducer total variable capacitance. The variable resistors (R_1_, R_2_) are used to calibrate the measuring bridge. The bridge unbalance is rectified and amplified by an operational amplifier.

The electronic adapter, Figure 4, was conceived in order to ensure a good proportionality between equivalent capacity C_Σ_ and the transducer output voltage (V_out_):

(10) $$V_{out}=k_{C}\cdot C_{\Sigma }=k_{C}\cdot \left[k_{a}\left(\varepsilon_{l}-\varepsilon_{g}\right)\cdot h+k_{a}\cdot \varepsilon_{g}\cdot H\right]=k_{h}\cdot h+V_{0}$$

with $k_{h}=k_{C}\cdot k_{a}\left(\varepsilon_{l}-\varepsilon_{g}\right)$, $V_{0}=k_{C}\cdot k_{a}\cdot \varepsilon_{g}\cdot H$, neglecting the parasitic capacity (C_p_). In addition, the bias (V_0_) is compensated by the variable resistors.

**Figure 5:**
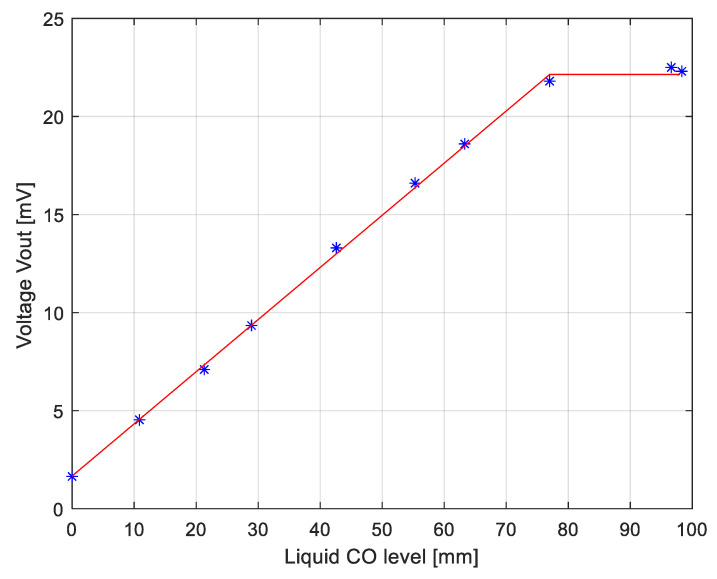
Liquid CO-level transducer: experimental data (stars) and theoretical results (solid line).

In Figure 5 are presented the experimental, measured results of the level transducer (marked with stars)—in the range of 0–78 mm of the CO liquid level—compared with the results given by the Equation (10), solid line. It is observed a good linearity, with the norm of residuals of 0.62685 and the R-square equal to 0.99, computed in Matlab^TM^. Outside this range, the transducer is saturated, but the present application do not need other values.

From constructive point of view, the transducer for liquid carbon-dioxide level in cryogenic isotope-separation column is an embedded component, maid of special stainless steel. The main cylindrical component of the capacitive transducer, insulated by the column body, is also made of stainless steel as a particular component of the column. The second part of the capacitor is a column-grounded body. An important issue solved here is the achievement of the connections from cylinder of the capacitor to the outside, with the proper technology of the column producer, due to very low working temperature in the column.

Using this sensor, some technical issues must be taken into account, detailed below.

In the thermal steady-state, the dimensions of the sensor are constant, so that the Equation (8) is valid. The transducer may be calibrated measuring the total capacity (C_Σ_)—or another signal, proportional to it.

In the thermal transient (in-rush, analysis, experiments, etc.) the dimensions change and generate some errors.

By in-rush phase, the internal capacitor armature is strongly cooled and its diameter decreases:

(11) $$D_{2}^{*}=D_{2}\cdot [1+\alpha \cdot (\theta_{op}-\theta_{e})]<D_{2}$$

where (*α*) is the thermal coefficient of dilatation (θ_op_) in the operation temperature and (θ_e_) is the environment temperature. The dielectric “thickness”

(12) $$d^{*}=\frac{D_{1}-D_{2}^{*}}{2}>d$$

increases and the total capacity decreases [20]:

(13) $$C_{\Sigma }^{*}=\frac{k_{a}}{d^{*}}(\varepsilon_{l}-\varepsilon_{g})\cdot h+\frac{k_{a}}{d^{*}}\cdot \varepsilon_{g}\cdot H+C_{p}$$

The indicated value of the level (h) is *smaller* than the real value, introducing the relative error:

(14) $$\left|\Delta \varepsilon_{1}\right|≅\frac{d^{*}-d}{d}≅\alpha \cdot \Delta \theta$$

For Δθ = 210 (°C) and *α* ≈ 23∙10^−6^ (°C)^−1^, Δε_1_ ≈ −0.483 (%).

For calibration, the sensor external wall is strongly cooled: the external capacitor armature contracts first, so that the dielectric thickness decreases:

(15) $$D_{1}^{*}=D_{1}\cdot [1+\alpha \cdot (\theta_{op}-\theta_{l})]<D_{2}$$

(16) $$d^{**}=\frac{D_{1}^{*}-D_{2}^{}}{2}>d$$

while the total capacitor value increases.

The indicated value of the level (h) is greater than the real value. The relative error may be approximated by the same equation.

### 3.2. Sensor for Liquid Nitrogen Level in Condenser

The goal of the second sensor is to offer information about the liquid nitrogen level in the condenser. Due to the very low temperatures, related to cryogenic techniques, the specific equipment must solve a series of major problems: stable behavior, without significant deformations, effective thermal insulation, etc. Different types of level transducers used in the field of cryogenic processes related to liquid nitrogen are known: carbon film resistors, hot wire transducers, cryo-diodes, ultrasonic or conventional capacitive transducers, etc. The choice of concrete solution depends primarily on the range of variation of the liquid nitrogen level. Ultrasonic transducers are used for level variations of centimeters, while for small variations—of the order of millimeters—are known applications using concentric capacitors with hot wire or carbon film. In all these mentioned constructive solutions, a series of disadvantages are noticed: low sensitivity and accuracy, high price, the need for complex electronic circuits for adaptation, hysteresis type nonlinear behavior due to the phenomena that appears in open vessels with liquid nitrogen, the intense superficial boiling being the main disturbing factor.

It is a great difference in the information processing given by the sensors of the liquid carbon monoxide level and of the liquid nitrogen level. The liquid carbon-monoxide sensor must generate a signal proportional to the small level (about 8 cm) in the boiler, the information about this level being crucial for the column operation supervision. In order to maintain the liquid nitrogen at a desired value, for the automatic control system is more useful a sensor with nonlinear characteristic, not available on the market. The nitrogen level range in column is about 75 cm and the control system must maintain the desired level anywhere in this range with an accuracy of 3 mm. Choosing an ON–OFF level control system based on the electromagnetic valve, the whole system is drastically simplified with the proposed nonlinear sensor. The solution is given by a sensor different from the previous one, which generates useful signal only in a small level range. This sensor is presented in detail in patent proposal [29].

**Figure 6:**
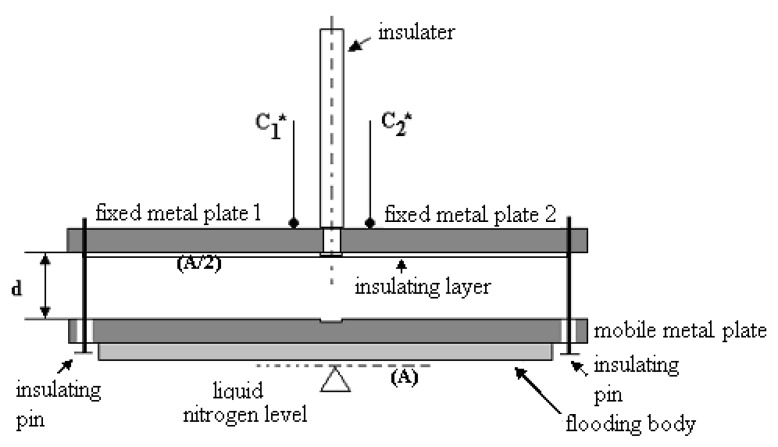
Section of capacitive-level sensor.

The proposed sensor is based on two parallel horizontal plates with area (A/2), each with semicircular shape, placed in the same plane, Figure 6.

The superior plate with area (A) is fixed on the vertical insulator, while the inferior plate can move in vertical direction, together with a floating body. Based on the equation of the plane condenser capacity [30]:

(17) $$C=\varepsilon \cdot \frac{A}{d}$$

the capacity changes with the nitrogen level evolution due to the variation of both parameters: permittivity (εlεg) and distance (d). Since the permittivity ratio in the case of isotope separation is (εlεg=1.45), the capacity variation with the nitrogen level is relatively small. For the proposed sensor also changes the distance (d) between capacitor plates with the nitrogen level, which generates much more variation of the sensor capacity. In accord to Figure 6, the distance (d) varies in the domain 0.5–6 (mm). The plates of nonlinear transducers for the liquid nitrogen level in the condenser are made of typical one-side plates for printed electronic circuits. The electrical connections are achieved of copper with Teflon insulation. The sensor support is made of insulated carbon fiber bare.

**Figure 7:**
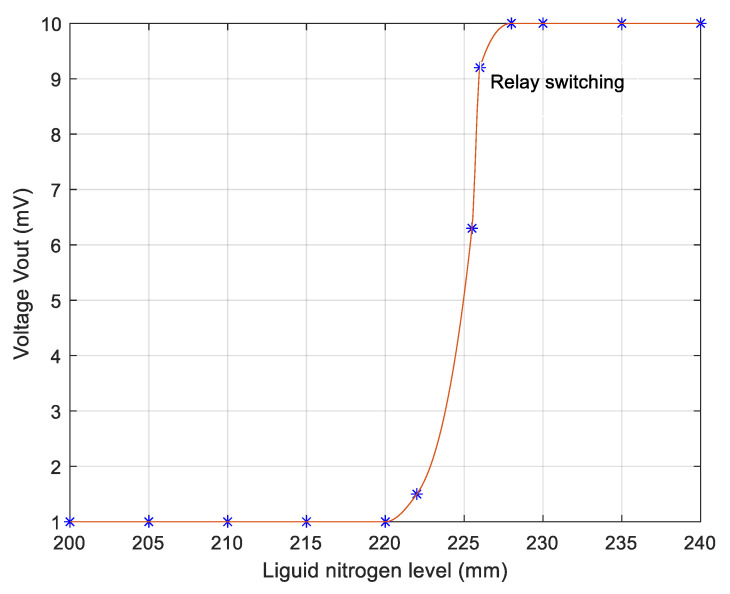
Liquid nitrogen level transducer: experimental data (stars) and theoretical results (solid line).

The corresponding electrical circuit is similar with the circuit presented in Figure 4, with the difference of the final element, which is now an electromechanical relay used to control the electromagnetic valve. The output voltage is the voltage over the relay coil. The relay contacts switch at a voltage of about V_out_ = 8 V. Unlike the usual CO-level sensor in the boiler which must exhibit a good linearity, the liquid nitrogen sensor has a nonlinear characteristic, typical for accurate level control systems. In Figure 7 is presented with solid line the theoretical dependence between output voltage over the relay coil and the liquid nitrogen level, while the stars represent the experimental data, exhibiting good accuracy. The corresponding mean squared error is 0.012.

Unlike the usual linear character of commercially available level transducers, the presented liquid nitrogen-level sensor can be used to maintain the controlled nitrogen level in a very narrow band due to the nonlinear characteristics.

### 3.3. Sensor for Electrical Power in the Boiler

Another essential variable which controls the column operation is the thermal power developed in the boiler. The thermal power is controlled by the electrical power generated by a dedicated resistor (heating element). The third transducer proposed in this study gives information about the electrical power (P_el_) dissipated in the boiler.

Different types of analog transducers are known for measuring power in direct current. They differ primarily in how the multiplication (mathematical product) is achieved between the voltage and current. Among the methods of principle are the use of the Hall effect, the use of square signal generators and, respectively, the use of the pulse width modulation (PWM) [30,31]. All the variants mentioned above have a number of disadvantages: complicated and in some cases, voluminous electronic circuits, sensitivity to ambient temperature, low reproducibility and nonlinear behaviors. The technical problem solved by the proposed solution is to make a transducer with a simplified structure electronic circuit, with reduced sensitivity to various external factors (e.g., variation of ambient temperature, variation of supply voltages), with extended linear operating area and high accuracy. The transducer is described in detail in patent [32].

**Figure 8:**
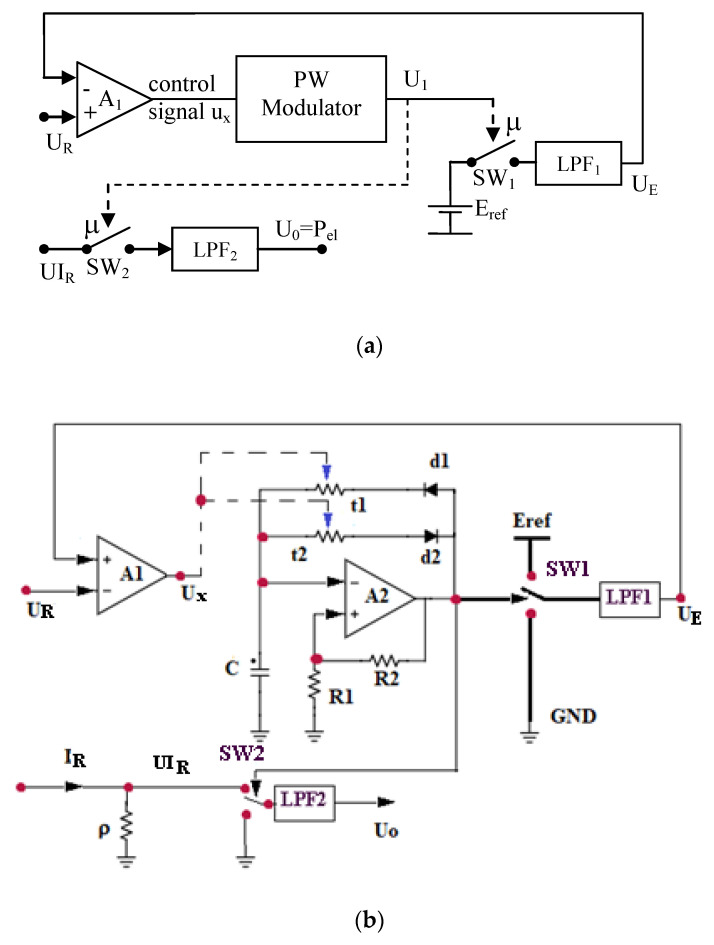
Proposed electrical power transducer: (**a**) Principle of operation; (**b**) electric circuit.

In the separation process it is recommended a DC power supply for the boiler resistor in order to avoid the electromagnetic interferences (EMI). The proposed transducer is based on the pulse–width-modulator (PWM) principle, implemented with optocouplers as variable resistors, in closed loop, as it is schematically presented in Figure 8a. Due to the variations of the heating element resistance, the power value is obtained using the product (P_e_ = U_R_∙I_R_), where (U_R_) is the voltage, (I_R_) is the current related to the heating resistor and (P_e_) is a power proportional with (P_el_). The current (I_R_) is sensed by a usual shunt (ρ) [32], which gives a voltage (UI_R_) proportional to the current (I_R_).

(18) $$UI_{R}=\rho \cdot I_{R}$$

The operating principle of the transducer, Figure 8b, is based on the modification of the duty ratio (μ) by the PWM modulator, two electronic switches (SW1, SW2) realized with transistors and two low pass filters (LPF1) and (LPF2). To generate the appropriate value of the duty ratio (μ), a flip flop circuit is used based on amplifier (A2) with positive feedback through (R_1_) and (R_2_), but also with negative feedback using diodes (d1, d2), controlled equivalent resistances (r1, r2) and capacitor (C). Equivalent resistors correspond to two bipolar transistors from two interconnected optocouplers. The variation of the resistors (r1, r2) leads to the variation of the collector current that contributes to the charging/discharging of the capacitor (C), thus modifying the duty ratio (μ).

The “PWM modulator” generates a rectangular wave (U_1_) characterized by the duty ratio (μ) [32]:

(19) $$\mu =k_{PWM}\cdot u_{x}$$

where the voltage (u_x_) is the output error of the high gain amplifier (A_1_):

(20) $$u_{x}=A_{1}\left(U_{R}-U_{E}\right)$$

Here (U_E_) is the value of the low-pass filter (LPF_1_) output signal. The switch (SW_1_) is operated with the duty ratio (μ), so that [32]:

(21) $$U_{E}=\mu \cdot E$$

where (E) is the reference supply voltage for the (LPF_1_).

If A_1_ is a high gain amplifier, for finite values of (u_x_) results the equation:

(22) $$U_{R}\approx U_{E}=\mu \cdot E$$

so that the duty ratio is proportional with (U_R_) and inverse proportional with (E):

(23) $$\mu \approx \frac{1}{E}\cdot U_{R}$$

The same duty ratio (μ) drives the switch (SW_2_). The input of the low pass filter (LPF_2_) is the voltage (UI_R_) over the shunt (ρ) and the average value of the filter output voltage (U_0_) is:

(24) $$U_{0}=\mu \cdot UI_{R}$$

Using the Equation (24), results:

(25) $$U_{0}=\mu \cdot UI_{R}=\frac{1}{E}\cdot U_{R}\cdot UI_{R}=\frac{1}{E}\cdot U_{R}\cdot \rho \cdot I_{R}=k\cdot P_{e}$$

with k=ρE.

The output voltage (U_0_) proportional with (P_el_) gives information about the electrical power dissipated in the boiler:

(26) $$P_{e}=\frac{1}{k}\cdot U_{0}$$

In this study, is presented a particular version with U_R_ = (2;20) V_DC_, UI_R_ = (0.4;4) V_DC_ and U_0_ = (0.08;8.75) V_DC_, 1/k = 4.57. The value of the load resistor is R_L_ = 10 Ω and of the shunt is ρ = 2 Ω.

From constructive point of view this power transducer is based on usual electronic components: transistors, optocouplers, etc. The main advantage is the avoidance of the multiplying component (analog or digital) as main source of errors in order to generate the power signal.

**Figure 9:**
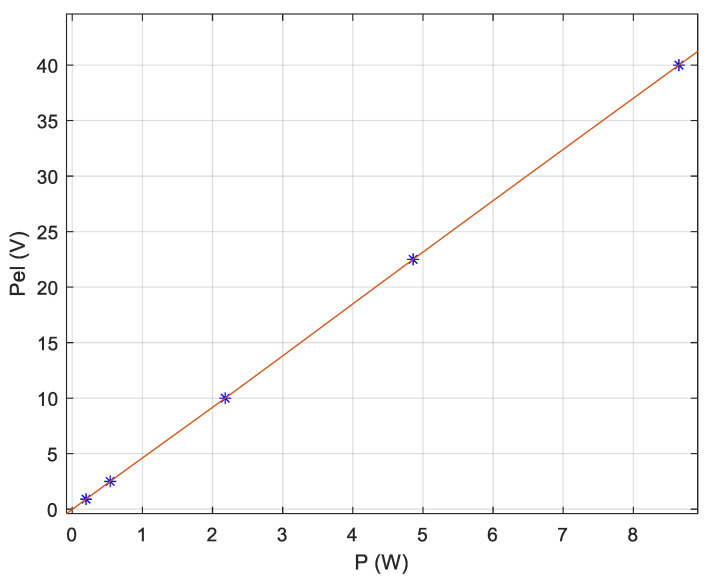
Static diagram of the electrical power transducer.

The static diagram of the electrical power sensor is given in Figure 9. Based on the well-known “volt–amperometric method” [31], with high precision voltmeter and ammeter (class 0.2) were measured: the voltages (U_R_), (UI_R_) and (U_0_) and the current (I_R_). The theoretic characteristic computed using the equation $P_{e}=k\cdot U_{R}\cdot I_{R}$, is represented with solid line in Figure 9. With stars are represented some measured output values of the power transducer. The maximum error was observed by the superior range limit, where the computed value is 8.75 V, the measured value is 8.65 V, resulting the absolute error of 0.1 V and the relative error of 1.14%. For the isotope-separation column operation, it is considered a good transducer quality [20].

## 4. Results and Conclusions

The three sensors designed for cryogenic isotope-separation columns were built together with the corresponding electronic elements.

**Figure 10:**
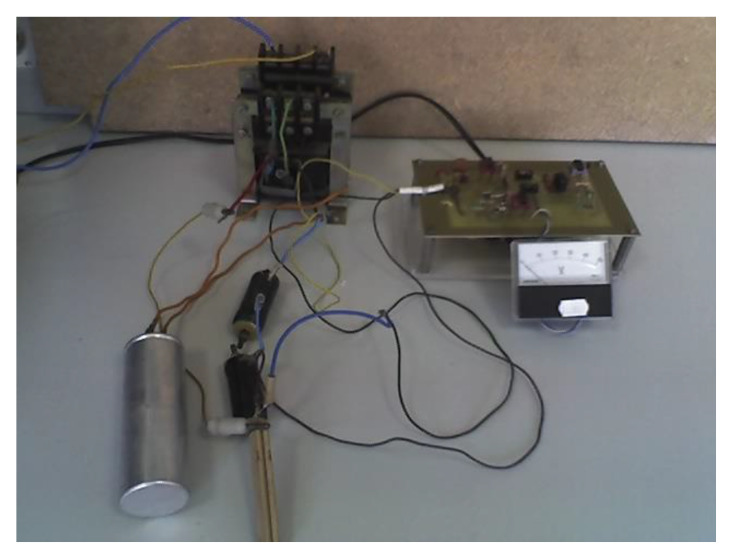
Liquid CO-level sensor: experimental version.

The capacitive carbon monoxide-level sensor is based on the difference between the coefficients of the dielectric permeability in liquid and gaseous phase. The electronic adapter was conceived in order to ensure a good proportionality between equivalent capacity of the sensor and the transducer output voltage. The experimental device is presented in Figure 10. The proposed transducer was tested in the range of 0–78 mm of the liquid CO level, highlighting proper operation.

**Figure 11:**
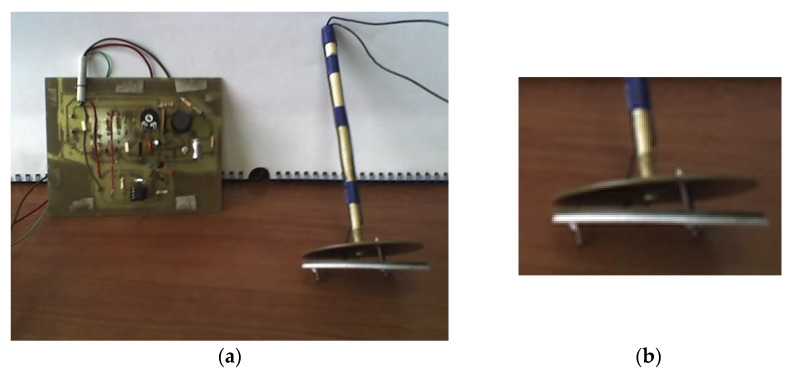
Liquid nitrogen-level sensor. (**a**) Experimental version; (**b**) zoom in on coplanar plates.

The nitrogen-level sensor consists of two coplanar metal plates, which are the fixed electrodes of the capacitive transducer and a metal plate insulated from the two upper plates and which can slide due to a float fixed to the lower metal plate, Figure 11. The signal from the capacitive-level sensor is processed by an electronic circuit adapted to different uses.

**Figure 12:**
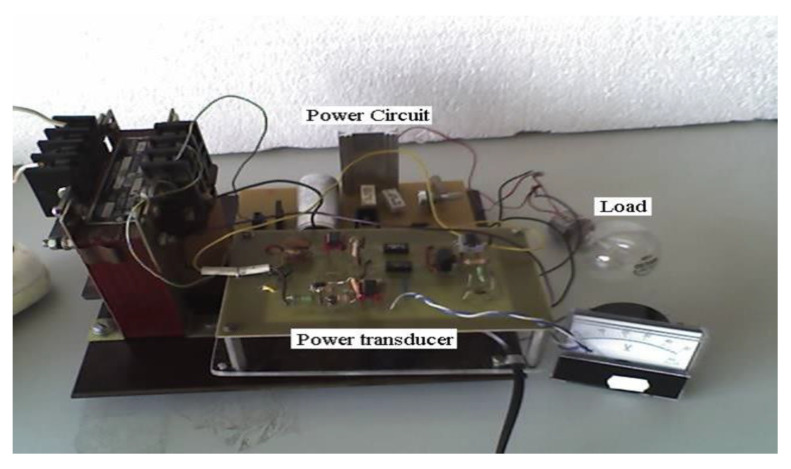
Electrical power transducer: experimental version.

The electrical power transducer, according to the patent, realizes in an original way the dependence between the duty ratio and the measuring circuit voltage. Using a closed loop negative feedback, the output signal of the transducer provides a voltage proportional to the power, Figure 12.

The main novelty of the proposed electrical power transducer is that improves the stability, reproducibility of measurements and measurement accuracy, using the (PWM) modulator closed in a negative feedback control loop.

The testing and calibration strategy was established and accomplished in National Institute for Research and Development of Isotopic and Molecular Technologies from Cluj-Napoca using dedicated equipment.

The transducers are reproducible (errors of reproducibility smaller than 2%) and will be used in automatic control of the equipment operation.
